# Evaluating STAT5 Phosphorylation as a Mean to Assess T Cell Proliferation

**DOI:** 10.3389/fimmu.2019.00722

**Published:** 2019-04-05

**Authors:** Michael Bitar, Andreas Boldt, Marie-Theres Freitag, Bernd Gruhn, Ulrike Köhl, Ulrich Sack

**Affiliations:** ^1^Medical Faculty, Institute of Clinical Immunology, University of Leipzig, Leipzig, Germany; ^2^Department of Pediatrics, Jena University Hospital, Jena, Germany; ^3^Hannover Medical School, Institute of Cellular Therapeutics, Hannover, Germany; ^4^Fraunhofer Institute for Immunology and Cell Therapy (IZI), Leipzig, Germany

**Keywords:** STAT5 activation, T cell proliferation, T cell activation, flow cytometry, CD25

## Abstract

Here we present a simple and sensitive flow cytometric—based assay to assess T cell proliferation. Given the critical role STAT5A phosphorylation in T cell proliferation, we decided to evaluate phosphorylation of STAT5A as an indicator of T cell proliferation. We determined pSTAT5A in T cell treated with either CD3/CD28 or PHA. After stimulation, T cells from adult healthy donors displayed a strong long-lasting phosphorylation of STAT5A, reaching a peak value after 24 h. The median fluorescence intensity (MFI) of pSTAT5A increased from 112 ± 17 to 512 ± 278 (CD3/CD28) (24 h) and to 413 ± 123 (PHA) (24 h), the IL-2 receptor-α (CD25) expression was greatly enhanced and after 72 h T cell proliferation amounted to 52.3 ± 10.3% (CD3/CD28) and to 48.4 ± 9.7% (PHA). Treatment with specific JAK3 and STAT5 inhibitors resulted in a complete blockage of phosphorylation of STAT5A, CD25 expression, and suppression of T cell proliferation. Compared with currently available methods, STAT5A phosphorylation is well-suited to predict T cell proliferation. Moreover, the method presented here is not very time consuming (several hours) and delivers functional information from which conclusions about T cell proliferation can be drawn.

## Introduction

The decision of T cells to start an appropriate activation- proliferation program upon encountering an antigen presented by an antigen presenting cell is a critical step of the adaptive immune reaction (1, 2).

**Figure 1:**
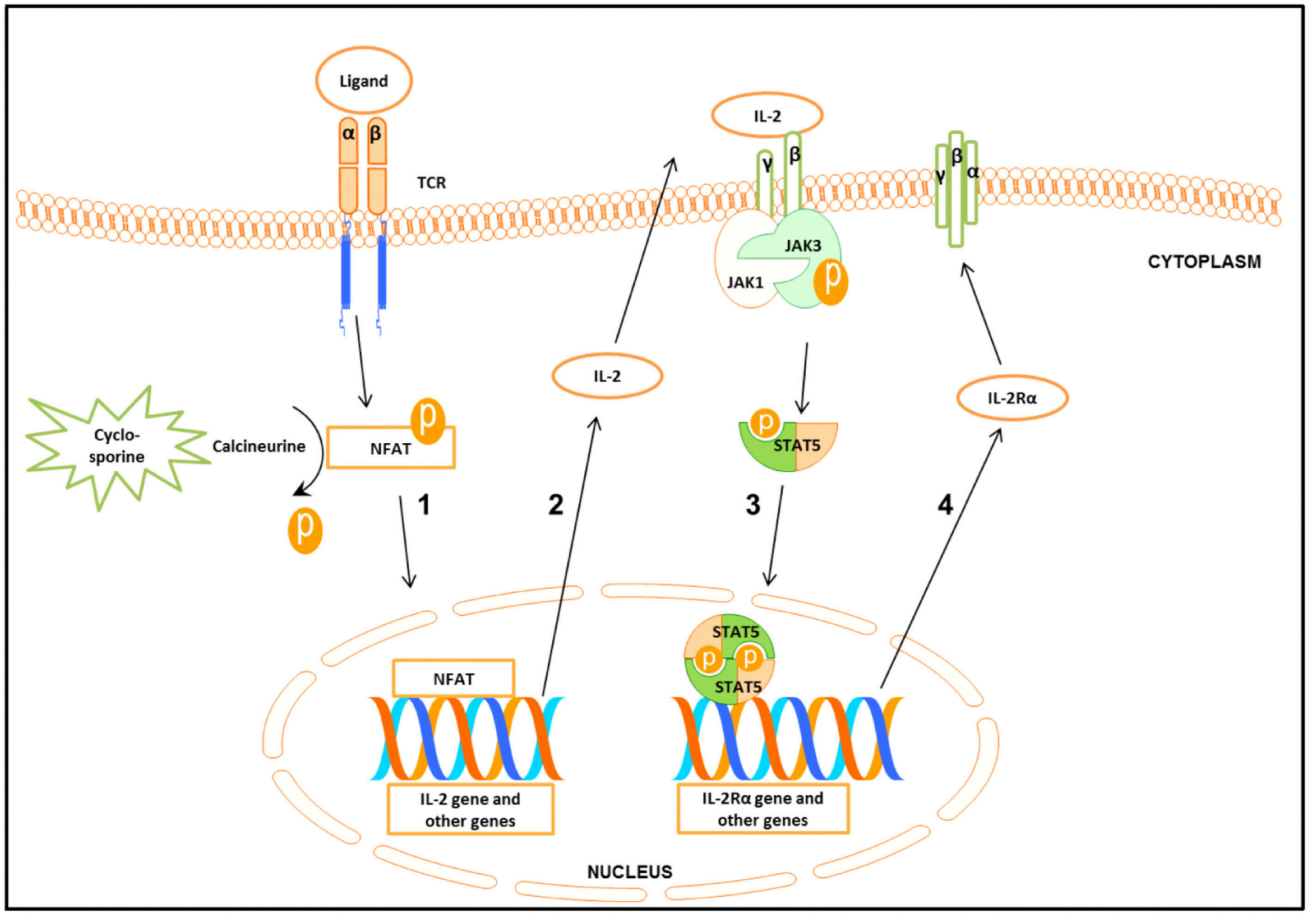
Schematic presentation of the signal cascade following T cells stimulation **(1)** Binding of the ligand to the T cell receptor (TCR) leads to the activation of nuclear factor of activated T cell (NFAT). Cyclosporine by inhibiting calcineurine prevents activation of NFAT. Activated NFAT translocates to the nucleus where it activates target genes including IL-2. **(2-3)** Binding of IL-2 to IL-2 receptorβγ (IL-2Rβγ) leads to the activation of Janus kinase 3 (Jak3) which in turn phosphorylates signal transducer and activator of transcription 5 (STAT5). Phosphorylated STAT5 forms homo- and heterodimers that translocate to the nucleus, leading to the transcription of target genes including the IL-2Rα gene. **(4)** IL-2Rα together with IL-2Rβγ forms the high affinity IL-2Rβ*γα*.

Following engagement of the T cell receptor (TCR), three transcription factors, namely nuclear factor of activated T cells (NFAT), nuclear factor kappa-light-chain-enhancer of activated B cells (NF-κB), and activator protein 1 (AP-1) will be activated (3, 4). The interaction between these molecules leads to the synthesis of important cytokines such as Interleukin−2 (IL-2) and Interferon gamma (IFN-γ) (5), as well as the up-regulation of Janus kinase 3 (Jak3) expression (Figure 1) (4, 6–8).

TCR stimulated production of IL-2 and other cytokines starts the cascade of signaling events, leading to the activation of Jak3, which in turn phosphorylates signal transducer and activator of transcription 5 (STAT5) (Figure 1) (8). STAT5 consists of two highly related proteins, STAT5A and STAT5B, which share over 90% identity and differ in their carboxyl (C)—terminus (9, 10). Both STAT5A and STAT5B regulated genes are involved in cell proliferation, survival, differentiation and apoptosis (10, 11).

Phosphorylated STAT5 (pSTAT5) translocates into the nucleus to regulate transcription of the target genes including the IL-2 receptor α (IL-2Rα) (CD25) (Figure 1) (5, 12–14), a prerequisite for the formation of the high affinity IL-2Rα*βγ* (12, 15, 16). The induction of the functional system composed of IL-2 and the high affinity IL-2R is critical for G1 progression and for mounting an effective immune response (Figure 1) (12, 17).

One standard procedure to quantify cellular immune responses to antigens is based on the measurement of cell proliferation (1, 2). Today, the assays are mainly carried out by the use of flow cytometry (FCM). One of the methods consists of serial halving of the fluorescence intensity of the vital dye (18). The current assays have many drawbacks including the need of bulk cultures and long incubation times (3–5 days). This is especially inconvenient when rapid diagnosis is desirable. Therefore, a fast and simple flow cytometric method enabling the early and reliable detection of lymphocyte entry into an activation program would be of great interest.

In this work, we asked whether phosphorylation of STAT5A is an appropriate candidate to predict the behavior of T cells upon activation. We established and validated a rapid, sensitive, flow cytometric based pSTAT5A assay to detect T cell proliferation. We showed that there was a strong correlation between the early CD3/CD28 or polyclonal mitogen phytohemagglutinin (PHA) induced STAT5A phosphorylation and T cells proliferation. Moreover, due to its simplicity and robustness, the flow cytometric based pSTAT5 assay is especially appropriate to rapidly assess primary immune deficiencies (PIDs) associated with STAT5 defects including autoimmune diseases, CD25 deficiency and T cells proliferation defects (11, 19–22).

## Methods and Material

### Collection of Blood Samples

Heparinized peripheral blood samples (7 ml) were taken from 19 adult healthy donors (median of age = 31), at the Institute of Clinical Immunology at the University of Leipzig. Additionally, we analyzed a blood from a patient selected by their clinical representations: anemia, clubfeet, and pancytopenia. Written informed consent was obtained from all included individuals. Sample collection and processing were completed according to the Medical Faculty, University of Leipzig standard operating guidelines and regulations.

### Isolation of PBMCs and Staining With Violet Proliferation Dye 450

Peripheral blood mononuclear cells (PBMCs) were isolated from fresh peripheral blood samples by density gradient centrifugation over Ficoll-Hypaque (Pan Biotech, Germany), as described previously (23, 24). PBMCs (1 ^*^ 10^7^ cells/ml) were diluted with phosphate-buffered saline (PBS, pH 7.2) (Gibco life Technologies, USA) and stained with Violet Proliferation Dye 450 (VPD450) (3 μM) (BD Biosciences) for 15 min at 37°C. Subsequently, PBMCs were washed and re-suspended in RPMI 1,640 containing 10% fetal bovine serum, penicillin (1 ^*^ 10^5^ mg/ml) and streptomycin (1 ^*^ 10^5^ mg/ml) (Gibco life Technologies, USA) and finally adjusted to 1 ^*^ 10^6^ cells/ml.

### Stimulation of PBMCs and Treatment With Specific Inhibitors

PBMCs (1 ^*^ 10^6^ cells/ml) were seeded into 48 well cell culture plates (5 ^*^ 10^5^ cells/well) at 37°C. After 2 h, PBMCs were stimulated with either CD3/CD28 (eBioscience, clones OKT3, CD28.2) (100 ng/ml) or with PHA (Sigma) (10 μg/ml).

Following pharmacological inhibitors: JAK3 inhibitor [JAK3i, 4-(4′-Hydroxyphenyl) amino-6, 7-dimethoxyquinazoline] (12 μM), STAT5 inhibitor [STAT5i, N′-((4-Oxo-4H-chromen-3-yl) methylene) nicotinohydrazide] (35 μM), Cyclosporin A (CsA) (500 nM) (Calbiochem, USA) or DMSO (0.07%) were added 2 h before stimulating the cells.

In parallel, cells were either cultured for 24 h to determine pSTAT5A and CD25 in T cells or for 72 h to determine T cell proliferation in a humidified atmosphere of 5% CO_2_ at 37°C.

### Determination of pSTAT5A and CD25 Expression in T Cells by Flow Cytometry

Cultured PBMCs were harvested after 24 h, pelleted by centrifugation, lysed and fixed by using “lyse and fix” buffer (BD Biosciences) and incubated at 37°C in a water bath for 12 min. The cells were centrifuged, the supernatant was discarded and the pellet was washed with 4 mL PBS.

The samples were permeabilized by using cold perm buffer III (1 ml) (BD Biosciences) and left on ice for 30 min. The pellet was washed three times with a fetal bovine serum stain buffer (FBS) (2 ml) (BD Biosciences) and finally re-suspended in 200 μl FBS. For flow cytometric analysis, the T cells were stained with PerCP-Cy™ 5.5 mouse anti-human CD3 (2.5 μL, clone UCHT1, BD Biosciences), PE mouse anti-human CD25 (5 μL, clone 2A3, BD Biosciences) Alexa Fluor 647 mouse anti human -STAT5A (10 μL, PY694, Clone 47/Stat5, BD Biosciences) and Alexa Fluor 647 mouse anti human FoxP3 (10 μl, clone 259D, c7, BD Biosciences). Mouse IgG1-k- Alexa Fluor 647 isotype control (10 μl, clone MOPC-21, BD Biosciences) was used for assessing the background staining of cells. After 1 h of incubation in the dark at room temperature, the cells were washed with 2 mL stain buffer, centrifuged and were suspended in 300 μL of stain buffer. To analyze STAT5A phosphorylation: based on the following gating strategy (1) forward scatter (FSC) vs. side scatter (SSC) and (2) CD3 vs. SSC, the T cells (CD3^+^) were separated (Supplementary Figure 1). Now, after clear separation of T cells from the non-T cells, 30,000 CD3^+^ T cells per sample were detected. The phosphorylation of STAT5A was calculated as median fluorescence intensity (MFI) in CD3^+^ T cells.

### Determination of T Cell Proliferation by Flow Cytometry

Cultured PBMCs were harvested after 72 h and washed with 2 mM EDTA PBS. The pellet was re-suspended in 200 μl PBS and stained with APC–H7 mouse anti-human CD45 (2.5 μl, clone 2D1, BD Biosciences) and FITC mouse anti-human CD3 (5 μl, clone SK7, BD Biosciences) at 37°C. After 15 min, cells were washed with 2 mL 2 mM EDTA PBS, centrifuged and suspended in 300 μL PBS.

To analyze T cell proliferation: based on two lymphocyte collection gates (1) FSC vs. SSC and (2) CD45 vs. SSC (mathematical connected by AND-operation) the T cells (CD3^+^) were separated in a third dot plot (CD3 vs. SSC). Now, the decrease of VPD450 dye intensity in proliferated CD3^+^ cells was measured, 50,000 CD3^+^ cells per sample were detected (Supplementary Figure 2). Data analysis was done using FlowJo.7.6.5 software (Ashland, OR, USA) (25).

### Cell Viability

7-Amino-Actinomycin D (7-AAD) staining was used to determine cell viability. 7-AAD is excluded by viable cells but can penetrate cell membranes of dead cells. 7-AAD (10 μl) (BD Biosciences) was added to pre-stained T cells (as described above) for 10 min, before cells were analyzed by flow cytometry.

### Flow Cytometric Analysis

FACSCanto II based flow cytometry was conducted to measure the samples as previously described (26, 27). Briefly, the system was set up with three lasers: a violet laser 405 nm, a blue laser 488 nm, and a red laser 647 nm. Prior to running samples, the instrument was calibrated using calibration beads (BD Biosciences). BD FACSDiva software was used for acquisition of events.

### Determination of IL-2 Production in PBMCs

PBMCs (1 ^*^ 10^6^ cells/ml) were cultured with PHA (Sigma) (10 μg/ml) for different times (12, 24, 48, 72 h). The cell supernatants were then collected and assayed for IL-2 by enzyme immunoassay (EIA; R&D system, Minneapolis, USA).

### Adaptation of Methods to DIN EN ISO 15189 Requirements

To exclude or diminish false positive or false negative results, the international standard DIN EN ISO 15189 recommended proceedings to fulfill highest requirements for the quality and competency of medical laboratories. This includes the validation of all data by performing intra-assay and inter-assay precision (28).

Note: in case of using two or more different FACS Canto, differences in technical adjustments among different devices leading to various results in the mean fluorescence and should be considered. Therefore, a transfer of the instrument setting among the devices has to be performed. Especially the voltage power in each channel in all devices should be equilibrated by the use of calibrate beads to get comparable MFI signals.

### Statistical Analysis

The statistical analysis was performed using the Graph Pad Prism 5 software (Graph Pad Prism software, Inc., San Diego, CA, USA). Curves were evaluated by the non-parametric Friedman test. The adjusted *P*-values were deemed by Wilcoxon's test (ns not significant, ^*^P ≤ 0.05; ^**^P ≤ 0.01; ^***^P ≤ 0.001). Correlations were calculated with Spearman's correlation coefficient.

## Results

### Immunodeficiency Is Accompanied by a Diminished Proliferation of T Cells and Down Regulation of pSTAT5A

**Figure 2:**
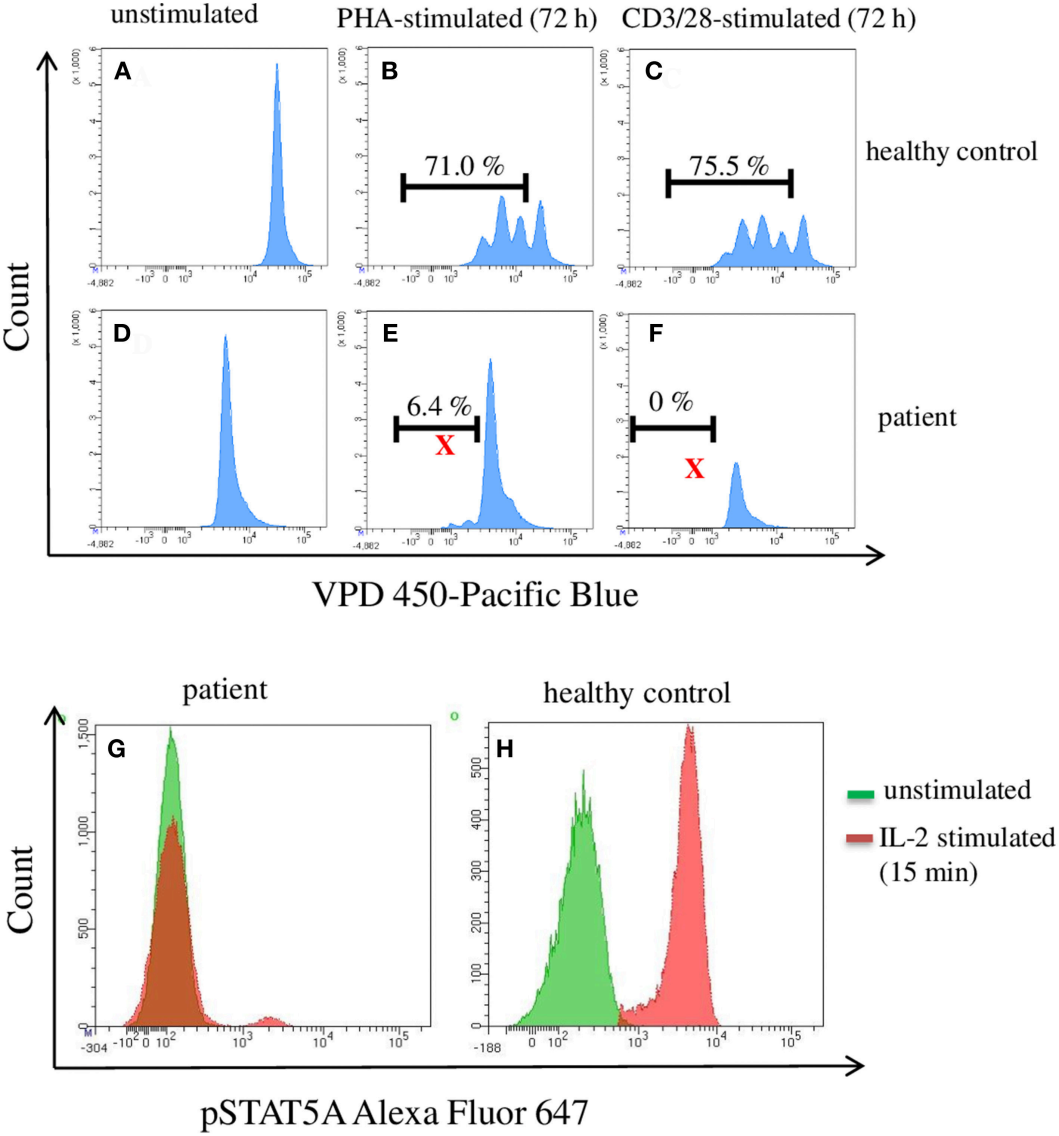
Flow cytometric analysis of pSTAT5A and T cell proliferation in PBMCs from a patient suffering from a congenital pancytopenia. PBMCs (1 * 10^6^ cells/ml) were treated with PHA (10 μg/ml) **(B,E)**, CD3/CD28 (100 ng/ml) **(C,F)**, or with IL-2 (100 ng/ml) **(G,H)**. After 15 min and 72 h, IL-2 stimulated pSTAT5A **(G,H)** and T cells proliferation **(A–F)** were determined, respectively.

An infant (female, 2 months old) of healthy, non-consanguine parents exhibited clinical symptoms such as umbilical hernia and clubfeet. Laboratory examinations revealed congenital pancytopenia: 0% neutrophils, 0% thrombocytes, and 100% naïve T cells. Nor B- or NK-cells could not be detected. For analyzing the function/proliferation of the T cells, we stimulated with CD3/CD28 or PHA (72 h) to investigate proliferation and with IL-2 (15 min) to measure pSTAT5A. As shown in Figure 2, the phosphorylation of STAT5A was completely deficient in patient T cells accompanied by severely limited T cell proliferation. Trio-exome sequencing did not reveal any abnormalities and excluded genetic defects in: IKZF1, GATA2, SAMD9, and SAMD9L. Finally, 14 month after successful unrelated bone marrow transplantation, the patient is in a very good clinical condition. Based on these data, we investigated in how far phosphorylation of STAT5A is a valid parameter to predict proliferation of T cells.

### Concentration and Time Dependent STAT5 Phosphorylation and Proliferation

**Figure 3:**
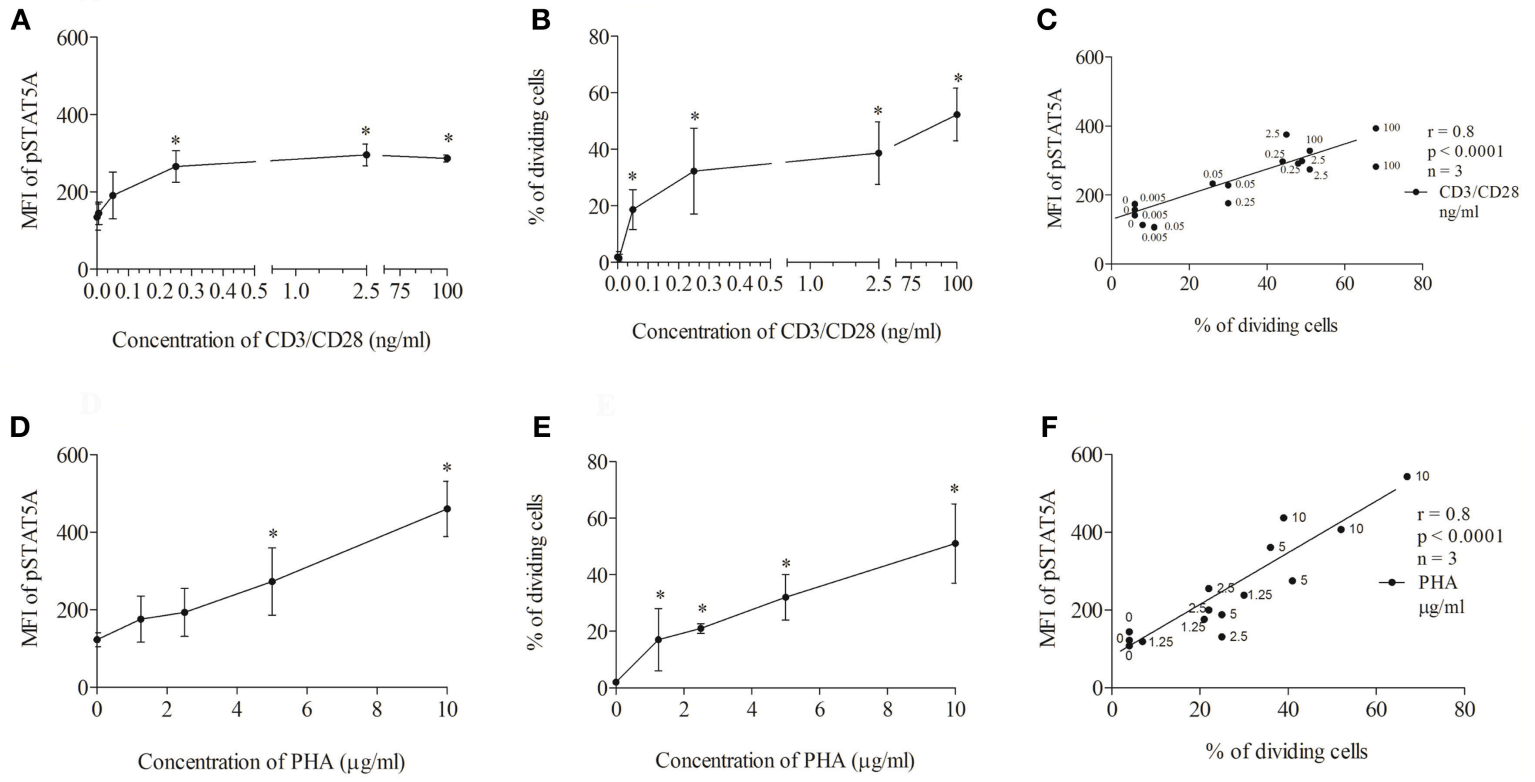
Concentration dependent correlation between the percentage of dividing cells and STAT5A phosphorylation. PBMCs (1 * 10^6^ cells/ml) were treated with CD3/CD28 (0, 0.005, 0.05, 0.25, 2.5, and 100 ng/ml) or PHA (0, 1.25, 2.5, 5, and 10 μg/ml). After 24 and 72 h, pSTAT5 **(A,D)** and proliferation **(B,E)** were determined, respectively. Correlation between the percentage of dividing cells (72 h) and STAT5A phosphorylation (24 h) dependent on the concentration of the stimulus **(C)** CD3/CD28, **(F)** PHA.*p < 0.05; r, Spearman's correlation coefficient; *n* = 3 independent experiments, MFI, median fluorescence intensity.

First, we tested the influence of different CD3/CD28 and PHA concentrations on STAT5A phosphorylation and T cell proliferation (Figure 3). We found that CD3/CD28 at a very low concentration of 0.25 ng/ml was sufficient to induce maximal phosphorylation of STAT5A (plateau phase) (Figure 3A) and that the percentage of dividing cells was highest at 100 ng/ml (Figure 3B). When stimulating the cells with PHA both pSTAT5A (Figure 3D) and the percentage of dividing cells (Figure 3E) steadily rose with increasing concentration of PHA up to 10 μg/ml. Thus in further experiments, we used CD3/CD28 at 100 ng/ml and PHA at 10 μg/ml.

As demonstrated in Figures 3C,F independent of the stimulus used a strong correlation [Spearman's correlation coefficient (r) = 0.8, *p* < 0.0001] could be observed between STAT5A phosphorylation and the percentage of dividing cells.

To determine the optimal time to analyze STAT5A phosphorylation, we performed a series of kinetics. We found that the CD3/CD28—induced phosphorylation of STAT5A reached a peak value after 24 h and that it declined thereafter (Supplementary Figure 3A). Peak values after the stimulation with PHA were reached between 12 and 24 h (Supplementary Figure 3B). Clearly, production of IL-2 was highest at 24 h (Supplementary Figure 3C). Thus, in further experiments, we analyzed pSTAT5A at 24 h.

### Inhibition of the JAK3/STAT5 Signal Cascade Leads to an Inhibition of pSTAT5A Signaling and T Cell Proliferation

**Figure 4:**
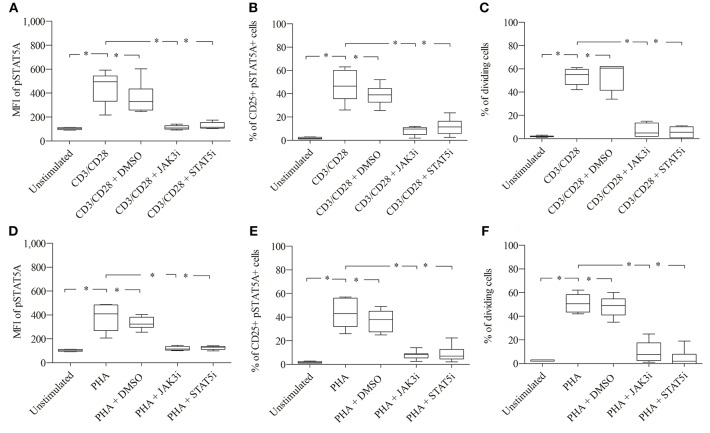
Role of pSTAT5A in T cell proliferation. CD3/CD28 or PHA stimulated T cells were treated either with DMSO (solvent control, 0.07 %), JAK3i (12 μM) or STAT5i (35 μM). After 24 h the MFI of pSTAT5 **(A,D)** the percentage of CD25^+^pSTAT5A^+^ cells **(B,E)** and after 72 h proliferation **(C,F)** were determined, The bold line inside each box plot shows the median, upper and lower lines indicate the maximum and minimum values, respectively (Curves were evaluated by the non-parametric Friedman test). *p < 0.05 (Wilcoxon's test); *n* = 6 independent experiments; MFI, median fluorescence intensity; Jak3i, Janus kinase 3 inhibitor; STAT5i, signal transducer and activator of transcription 5 inhibitor.

As seen in Figures 4A,B; Table 1, CD3/CD28 stimulated T cells showed a significant increase in both the MFI of pSTAT5A (from 103 ± 8 to 449 ± 134) and the percentage of CD25^+^ pSTAT5A^+^ cells (from 1.75 ± 0.7 to 46.6 ± 13.5 %) after 24 h.

**Table 1:** Summary of MFI of pSTAT5A, % of CD25^+^ pSTAT5A^+^cells and % of dividing cells, before and after treatment with JAK3 and STAT5 inhibitors in CD3/CD28 stimulated cells (*n* = 6).

**Control group**	**Unstimulated**	**CD3/CD28 (100 ng/ml)**	**CD3/CD28 (100 ng/ml) + DMSO (0.07 %)**	**CD3/CD28 (100 ng/ml) + JAK3i (12 μM)**	**CD3/CD28 (100 ng/ml) + STAT5i (35 μM)**
MFI of pSTAT5A^a^	103 ± 8	449 ± 134	358 ± 131	112 ± 17	127 ± 27
% of CD25^+^pSTAT5A^+^^b^	1.75 ± 0.7	46.6 ± 13.5	38.7 ± 8.7	8.3 ± 3.7	11.6 ± 7.2
% of dividing Cells^c^	2 ± 0.6	53.3 ± 7.3	53.8 ± 12	7 ± 5.7	5.5 ± 4.5

a *Median fluorescence intensity (MFI) of STAT5A after 24 h, calculated as mean ± SD*.

b *Percent of CD25^+^ pSTAT5A^+^ cells after 24 h, calculated as mean ± SD*.

c *Percent of dividing cells after 72 h, calculated as mean ± SD*.

Furthermore, after 72 h as determined by VPD450 dye staining 53.3 ± 7.3% of the T cells treated with CD3/CD28 proliferated (Figure 4C). Similar results were obtained upon activation with PHA (Figures 4D–F; Table 2)

**Table 2:** Summary of MFI of pSTAT5A, % of CD25^+^ pSTAT5A^+^cells and % of dividing cells, before and after treatment with JAK3 and STAT5 inhibitors in PHA stimulated cells (*n* = 6).

**Control group**	**Unstimulated**	**PHA (10 μg/ml)**	**PHA (10 μg/ml) + DMSO (0.07 %)**	**PHA (10 μg/ml) + JAK3i (12 μM)**	**PHA (10 μg/ml) + STAT5i (35μM)**
MFI of pSTAT5A^a^	103 ± 8	380 ± 111	331 ± 51	119 ± 15	125 ± 15
% CD25^+^pSTAT5A^+^^b^	1.75 ± 0.7	42.8 ± 11.4	36.5 ± 8.7	8.1 ± 3.6	8.9 ± 6.7
% of dividing cells^c^	2 ± 0.6	51 ± 7.6	48.2 ± 8.5	9.8 ± 9	4.5 ± 7

a *Median fluorescence intensity (MFI) of STAT5A after 24 h, calculated as mean ± SD*.

b *Percent of CD25^+^ pSTAT5A^+^ cells after 24 h, calculated as mean ± SD*.

c *Percent of dividing cells after 72 h, calculated as mean ± SD*.

When CD3/CD28 or PHA stimulated T cells were pre-incubated with STAT5i, the MFI of pSTAT5A was substantially abrogated or barely detectable [127 ± 27 (CD3/CD28), 125 ± 15 (PHA)] (Figures 4A,D; Tables 1, 2) and the percentage of CD25^+^ pSTAT5A^+^ cells was extremely low [11.6 ± 7.2% (CD3/CD28), 8.9 ± 6.7% (PHA)] (Figures 4B,E; Tables 1, 2). Importantly, inhibition of phosphorylation of STAT5A was associated with a suppression of dividing T cells [5.5 ± 4.5% (CD3/CD28), 4.5 ± 7 % (PHA)] (Figures 4C,F; Tables 1, 2) after 72 h.

Similar results were obtained upon inhibition with JAK3i (Figures 4A–F; Tables 1, 2)

**Figure 5:**
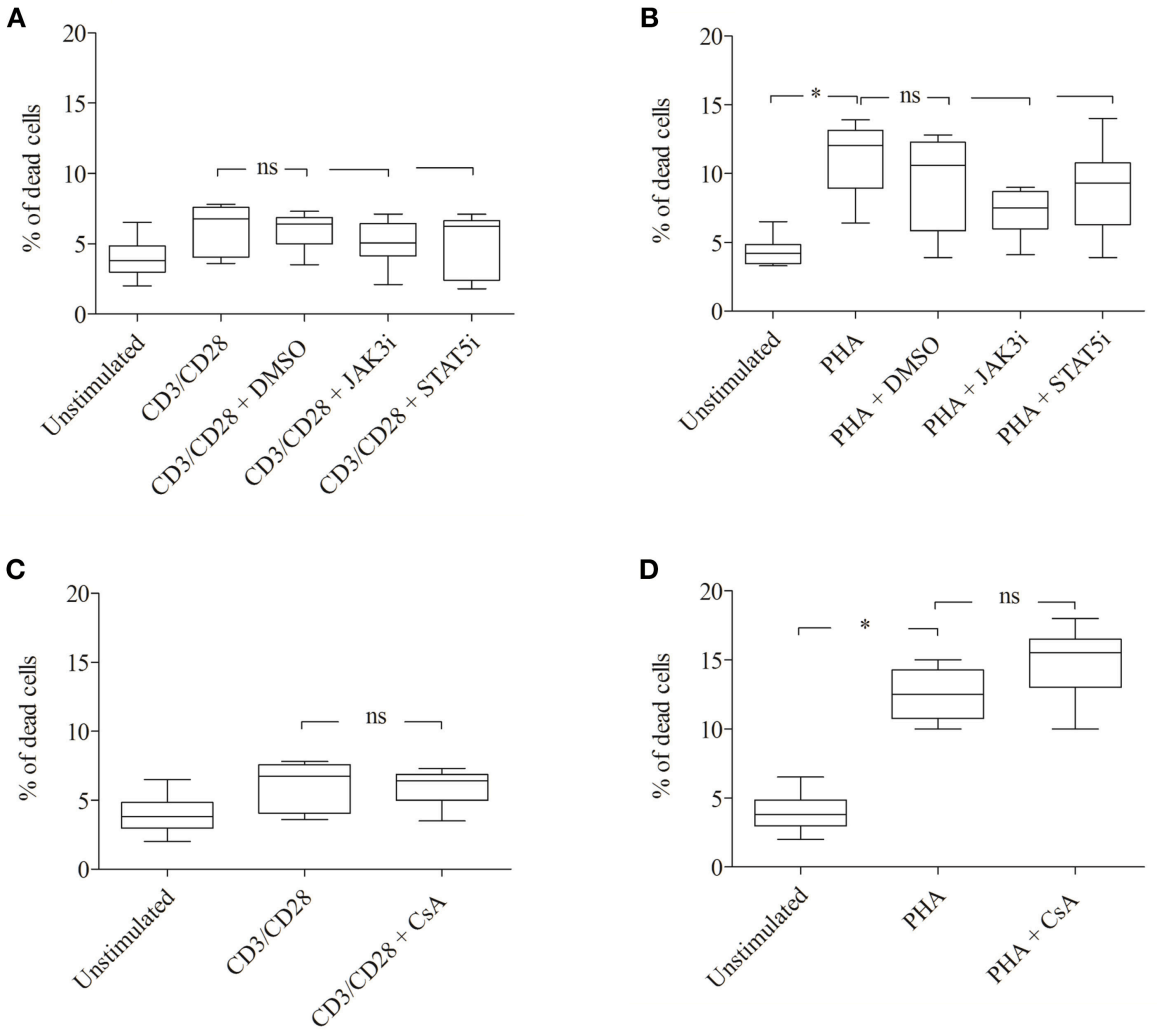
Effect of JAK3, STAT5 inhibitors and cyclosporine (CsA) on cell viability. CD3/CD28 or PHA stimulated T cells were treated with DMSO (0.07 %), JAK3i (12 μM), STAT5i (35 μM) **(A,B)** or CsA (500 nM) **(C,D)** for 72 h. The percentage of dead cells was calculated after 7-AAD staining. The bold line inside each box plot shows the median, upper, and lower lines indicate the maximum and minimum values, respectively (Curves were evaluated by the non-parametric Friedman test). Ns, no significant; *p < 0.05 (Wilcoxon's test); *n* = 6 independent experiments; Jak3i, Janus kinase 3 inhibitor; STAT5i, signal transducer and activator of transcription 5 inhibitor.

The inhibitory effect of JAK3i or STAT5i on proliferation was not due to an increased cytotoxicity, the percentage of dead cells hardly changed after 72 h of treatment (Figures 5A,B). To test whether the CD25^+^ cell population contained any regulatory T cells, we stimulated the cells with PHA or CD3/CD28 and measured the expression of the transcription factor FOXP3. Almost all CD25^+^ cells were FOXP3^−^ cells, the percentage of CD25^+^ FOXP3^+^ cells was low not exceeding 5–8% (data not shown).

### Inhibition of IL-2 Gene Transcription Leads to a Decrease of pSTAT5A Signaling and T Cell Proliferation

To examine the role of IL-2 in STAT5A phosphorylation, we used CsA an inhibitor of calcineurine, which blocks the NFAT activity (3). After antigen recognition by the TCR, NFAT binds to the promoter region of the IL-2 gene leading to its transcription.

**Figure 6:**
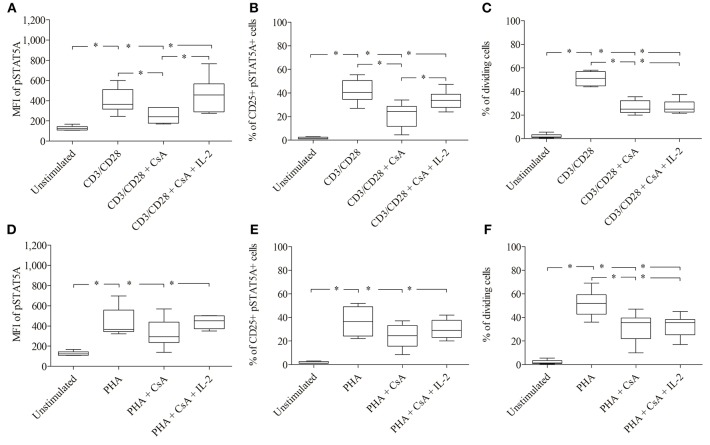
Inhibition of IL-2 transcription by Cyclosporine (CsA) correlates with an inhibition of JAK3/STAT5 mediated signal transduction and proliferation. CD3/CD28 **(A–C)** or PHA **(D–F)**—stimulated T cells were either treated with CsA (500 nM) or with CsA (500 nM) + IL-2 (100 ng/ml). After 24 h the MFI of pSTAT5 A **(A,D)**, the percentage of CD25^+^ pSTAT5A^+^ cells **(B,E)** and after 72 h proliferation were determined **(C,F)**. The bold line inside each box plot shows the median, upper and lower lines indicate the maximum and minimum values, respectively (Curves were evaluated by the non-parametric Friedman test). *p < 0.05 (Wilcoxon's test); *n* = 6 independent experiments; MFI, median fluorescence intensity.

As shown in Figure 6; Table 3 treatment with CsA led to a substantial decrease of MFI values of pSTAT5 (from 398 ± 123 to 249 ± 74) in CD3/CD28 and (from 435 ± 143 to 325 ±144) in PHA stimulated cells (Figures 6A,D; Table 3). This decrease was associated with a low percentage of CD25^+^ pSTAT5A^+^ cells stimulated with CD3/CD28 (from 41.5 ± 9.9 to 21.3 ± 10.9%) or with PHA (from 36.6 ± 11.9 to 24 ± 10%) (Figures 6B,E; Table 3). The number of proliferating T cells was reduced [from 50.9 ± 5.7 to 26.5 ± 5.8% (CD3/CD28), from 51.6 ± 11.1 to 31.8 ± 12.6% (PHA)] (Figures 6C,F; Table 3).

**Table 3:** Summary of MFI of pSTAT5A, % of CD25^+^ pSTAT5A^+^cells and % of dividing cells before and after treatment with Cyclosporine (CsA) in CD3/CD28 and PHA stimulated cells (*n* = 6).

**Control group**	**Unstimulated**	**CD3/CD28 (100 ng/ml)**	**CD3/CD28 (100 ng/ml) + CsA (500 nM)**	**CD3/CD28 (100 ng/ml) + CsA (500 nM) + IL- 2 (100 ng/ml)**	**PHA (10μg/ml)**	**PHA (10μg/ml) + CsA (500 nM)**	**PHA (10 μg/ml) + CsA (500 nM) + IL- 2 (100 ng/ml)**
MFI of pSTAT5A^a^	128 ± 22	398 ± 123	249 ± 74	458 ± 176	435 ± 143	325 ± 144	438 ± 64
% CD25^+^pSTAT5A^+^^b^	1.6 ± 0.8	41.5 ± 9.9	21.3 ± 10.9	33.9 ± 7.9	36.6 ± 11.9	24 ± 10	30 ± 8.3
% of dividing cells^c^	2 ± 1.8	50.9 ± 5.7	26.5 ± 5.8	26.8 ± 5.7	51.6 ± 11.1	31.8 ± 12.6	32.8 ± 9.4

a *Median fluorescence intensity (MFI) of STAT5A after 24 h, calculated as mean ± SD*.

b *Percent of CD25^+^ pSTAT5A^+^ cells after 24 h, calculated as mean ± SD*.

c *Percent of dividing cells after 72 h, calculated as mean ± SD*.

Importantly, the phosphorylation of STAT5A and the percentage of CD25^+^ pSTAT5A^+^ of CsA—treated T cells (Figures 6A–E), but not the proliferation (Figures 6C,F; Table 3) was rescued by adding exogenous IL-2 (100 ng/ml). We could exclude that the inhibitory effect of CsA on T cell proliferation was due to a loss of cell viability as assessed 72 h after treatment (Figures 5C,D).

### Validation of STAT5A Phosphorylation in Healthy Donors to DIN EN ISO 15189 Requirements and for Use in Diagnostic Application

**Figure 7:**
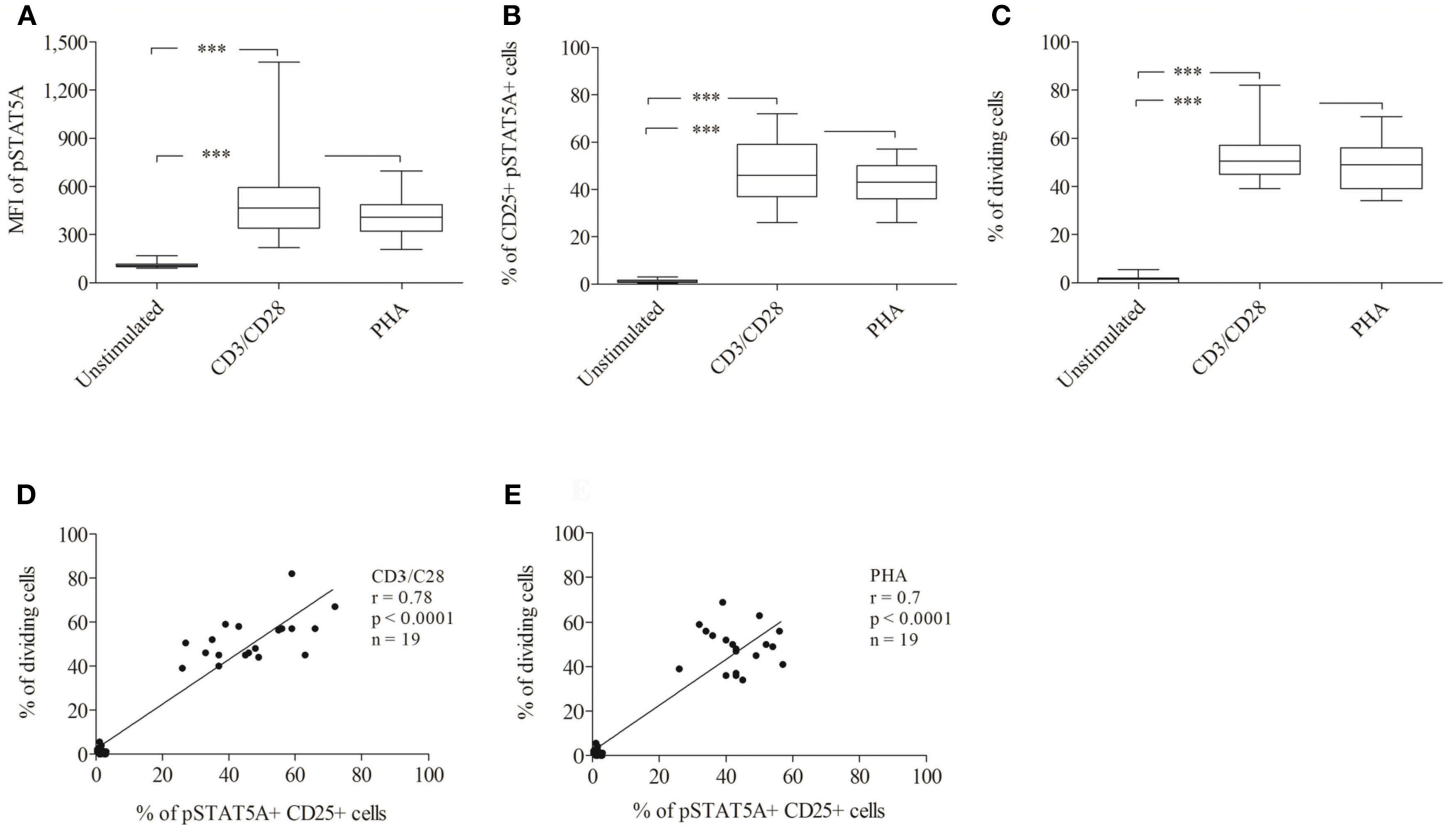
Analysis of STAT5A phosphorylation and percentage of CD25^+^ pSTAT5A^+^ cells and dividing cells by flow cytometry (*n* = 19). T cells were stimulated with CD3/CD28 or PHA. After 24 h the MFI of STAT5A **(A)** the percentage of CD25^+^ pSTAT5^+^ cells **(B)** and after 72 h proliferations **(C)** were determined. The correlation between the percentage of dividing cells (72 h) and percentage of CD25^+^ pSTAT5A^+^ cells (24 h) **(D)** CD3/CD28, **(E)** PHA. The bold line inside each box plot shows the median, upper and lower lines indicate the maximum and minimum values, respectively. ***p < 0.001 (Wilcoxon's test); r, Spearman's correlation coefficient; MFI, median fluorescence intensity.

To establish a rapid flow cytometric assay to evaluate STAT5A phosphorylation and to provide reference values for healthy adult controls, we analyzed the MFI of pSTAT5A and proliferation of T cells simultaneously (*n* = 19). In both, CD3/CD28 or PHA treated cells, the MFI of pSTAT5A was strongly increased [from 112 ± 17 to 512 ± 278 (CD3/CD28), 413 ± 123 (PHA)] (Figure 7A; Table 4). The percentage of CD25^+^ pSTAT5A^+^ cells was significantly up-regulated and the amount of CD25^+^ pPSTAT5A^+^ cells (24 h) correlated with the amount of dividing cells (72 h) (Figures 7D,E).

**Table 4:** Summary of MFI of pSTAT5A, % of CD25^+^ pSTAT5A^+^ cells and % of dividing cells in CD3/CD28 or PHA stimulated cells for healthy adult controls (*n* = 19).

**Control group**	**Unstimulated**	**CD3/CD28 (100 ng/ml)**	**PHA (10 μg/ml)**
MFI of pSTAT5A^a^	112 ± 17	512 ± 278	413 ± 123
% of CD25^+^pSTAT5A^+^^b^	1.3 ± 0.8	47.1 ± 13.2	42.6 ± 9
% of dividing cells^c^	1.5 ± 1.4	52.3 ± 10.3	48.4 ± 9.7

a *Median fluorescence intensity (MFI) of STAT5A after 24 h, calculated as mean ± SD*.

b *Percent of CD25^+^ pSTAT5A^+^ cells after 24 h, calculated as mean ± SD*.

c *Percent of dividing cells after 72 h, calculated as mean ± SD*.

Flow cytometric analysis revealed that the percentage of CD25^+^ pSTAT5A^+^ cells (47.1 ± 13.2% [CD3/CD28), 42.6 ± 9% (PHA)] after 24 h nearly mirrored the percentage of dividing cells after 72 h [52.3 ± 10.3% (CD3/CD28), 48.4 ± 9.7% (PHA)] (Figures 7B–E; Table 4).

Subsequently, to ensure that our analytical method is accurate, reproducible and precise, validation of our data included the definition of intra- and interassay precision values (28). A coefficient of variation up to 25 percent was considered tolerable and fulfilled the criteria of the International Standard EN ISO 15189.

## Discussion

Lymphocyte proliferation is commonly accepted as a reliable measurement of lymphocyte activation (1). The current assays have many drawbacks including the need of bulk cultures and long incubation times (3–5 days), which is inconvenient when rapid diagnosis is desirable. Therefore, we tried to establish a rapid, reliable method that can be used as an indicator of proliferation. We asked whether the phosphorylation of STAT5A is a trustworthy marker for predicting T cell proliferation.

We analyzed the effect CD3/CD28 or PHA on signaling pathways that are essential for T cell proliferation. Our results revealed that a moderate expression of pSTAT5A starts after a few hours of stimulation (6 h) by CD3/CD28 and PHA. It leads to an increased expression of CD25, a prerequisite to form the high affinity IL-2 receptor. Hence, IL-2 can achieve its biological effects such as inducing a sustained IL-2 dependent JAK3/STAT5 signal cascade, which leads to high phosphorylation of STAT5 (after 24 h) and cell proliferation (after 72 h). Our data displayed a strong correlation between these two events.

Interestingly, loss of phosphorylation of STAT5A by the specific inhibitors, that target the activity of STAT5 directly like STAT5i or indirectly like JAK3i led to down-regulation of the percentage of CD25^+^ pSTAT5A^+^ cells, accompanied by a diminished PHA or CD3/CD28—driven T cell proliferation.

In agreement with other studies (9, 12), these observations confirm that STAT5A activation, downstream of TCR signaling, plays an important role in inducing the transcription of the CD25 gene. This in turn leads to the formation of the high affinity IL-2R, which results in sustained prolonged JAK3/STAT5 activity and long-term CD25 up-regulation (9, 12).

We could not distinguish between STAT5A and STAT5B being involved in CD25 expression, because both proteins were affected by the inhibitors. Previously, Ivashkiv LB and Hu X 2004 (29) reported that T cells from STAT5A deficient mice displayed reduced proliferation rates secondary to a diminished expression of the IL-2Rα chain. Likewise, Kanai et al. (10) reported that STAT5B deficient patients have reduced numbers of natural killer cells, T cells and impaired IL-2 signaling. They showed that in humans the anti-apoptotic factor BCL2L1 is regulated by STAT5A, whereas FOXP3, and CD25 expression are regulated by STAT5B. Moreover, Lin and Leonard (14) described the importance of the functional cooperation of STAT5 with other transcription factors in regulating CD25 expression (14).

In order to clarify the role of IL-2 in the activation of the JAK3/STAT5 signal cascade, expression of CD25 and cell proliferation, we used CsA which is known to inhibit the nuclear entry of the transcription factor NFAT that is required for IL-2 gene transcription (30). The treatment with CsA substantially decreased the phosphorylation of STAT5A, the percentage of CD25^+^ pSTAT5A^+^ cells and the percentage of dividing cells.

Importantly, the phosphorylation of STAT5A and the percentage of CD25^+^ pSTAT5A^+^ in CsA treated cells was rescued by adding exogenous IL-2, but the percentage of dividing cells was not (after 72 h incubation). It is very well possible that CsA by inhibiting the phosphatase calcineurine displays effects that are not restricted to NFAT and thus interferes with mechanisms that are IL-2 independent.

Taken together, our data underline the general perception on TCR mediated proliferation. Signaling basically involves two steps, the first leading to the transcription of the IL-2 gene, the second starting with the activation of JAK3/STAT5 signaling which results in generating the high affinity IL2R. Thereafter, the irreversible decision to replicate DNA and proliferate is made.

In the current study, we showed a strong correlation between the STAT5A phosphorylation and percentage of dividing cells. According to our results including the data derived from the T cells of an immunodeficient patient, we suggest pSTAT5A as a new and rapid diagnostic flow cytometric marker. Additionally, we analyzed T cells from 19 healthy donors to identify a threshold for pSTAT5A values after stimulation with CD3/CD28 or PHA. However, each laboratory should determine and validate its own threshold value following appropriate validation procedures.

In conclusion, we introduced a rapid und straightforward flow cytometric—assay for the assessment of T cell proliferation, based on the staining of phosphorylated STAT5A. Our assay is unique because it identifies T cell proliferation by detecting immediate phosphorylation of STAT5A after stimulation. Because this method is rapid, robust and adaptable, it could be implemented for the measurement of T cells in patient's samples in a variety of clinical settings.

## Ethics Statement

The study protocol conformed to the ethical guidelines of the Declaration of Helsinki and was approved by the ethics committee of the University Leipzig (092/2002 and 151/2006). All donors gave written informed consent.

## Author Contributions

MB: generation and analysis of data, writing of the manuscript. AB: analysis and interpretation of data. M-TF: analysis of IL-2 production. BG: supervision of the patient. UK: validation of data. US: study supervision, design of the experiments.

### Conflict of Interest Statement

The authors declare that the research was conducted in the absence of any commercial or financial relationships that could be construed as a potential conflict of interest.

## Abbreviations

TCR: T cell receptor
NFAT: nuclear factor of activated T cells
IL-2: Interleukin−2
Jak3: Janus kinase 3
pSTAT5: phosphorylated signal transducer and activator of transcription 5
CD25: IL-2 receptor α
FCM: flow cytometry
PHA: phytohemagglutinin
PBMCs: Peripheral blood mononuclear cells
Jak3i: Janus kinase 3 inhibitor
STAT5i: signal transducer and activator of transcription 5 inhibitor
CsA: Cyclosporin A
MFI: median fluorescence intensity.
