# Supplementary material for: Evaluating STAT5 Phosphorylation as a Mean to Assess T Cell Proliferation
Source: Front Immunol. 2019 Apr 5;10:722. doi: 10.3389/fimmu.2019.00722 (PMC6460883; doi:10.3389/fimmu.2019.00722)
Supplement: Supplementary file 1 [file Data_Sheet_1.docx]

**
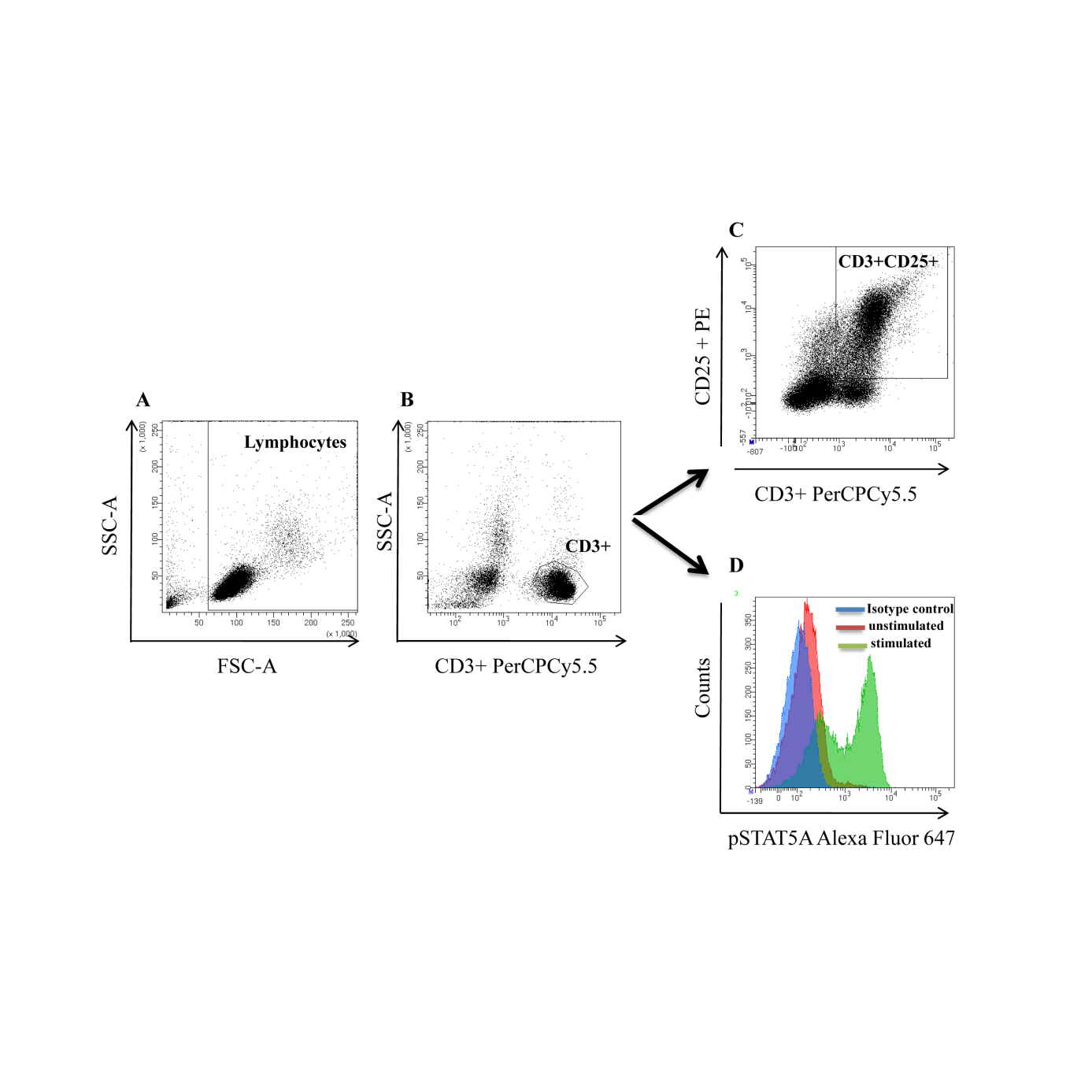
**

**Supplementary Figure 1.** Flow cytometric gating strategy to detect STAT5A phosphorylation and CD25+ T cells. Peripheral blood cells (1 * 10^6^ cells/ml) were stimulated with CD3/CD28 (100 ng/ml) or PHA (10 µg/ml). After 24 h, T cells were stained with PerCP-Cy TM 5.5 (mouse anti-human CD3), PE (mouse anti-human CD25), Alexa Fluor 647 (mouse anti human -STAT5A) according to the manufactures instructions (BD Biosciences, Heidelberg, Germany). Based on the following gating strategy forward scatter (FSC) vs. side scatter (SSC) and (2) CD3 vs. SSC **(B)**, the T cells (CD3+) were separated. CD25^+^ CD3^+^ cells were detected by dot plot as CD25 PE vs. CD3 PerCPCy5.5 **(C)** and pSTAT5A as histogram **(D).** Mouse IgG1-k- Alexa Fluor 647 was used as an isotype control for assessing the background staining of the cells.
