# Supplementary material for: Evaluating STAT5 Phosphorylation as a Mean to Assess T Cell Proliferation
Source: Front Immunol. 2019 Apr 5;10:722. doi: 10.3389/fimmu.2019.00722 (PMC6460883; doi:10.3389/fimmu.2019.00722)
Supplement: Supplementary file 2 [file Data_Sheet_2.docx]

**
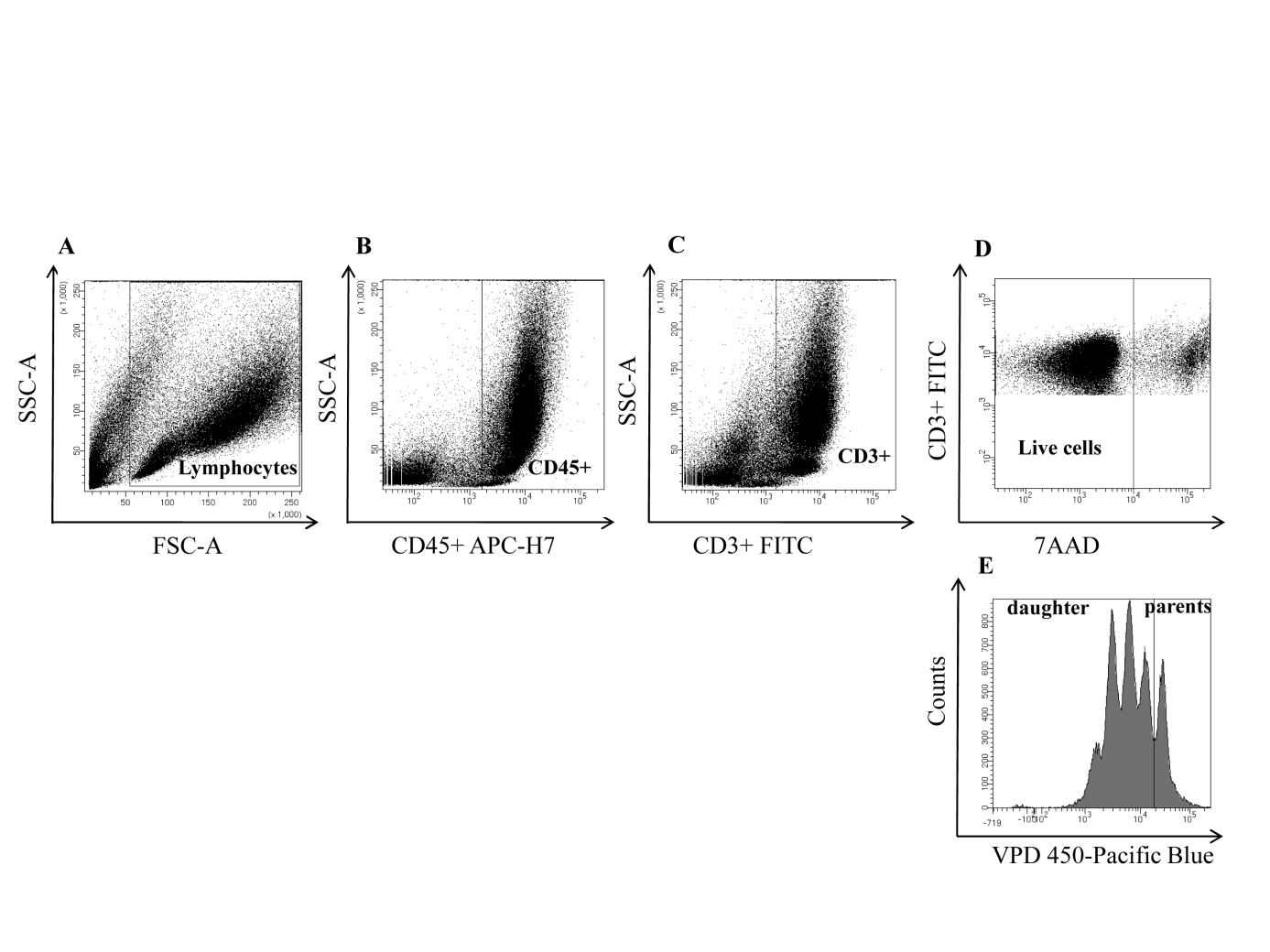
**

**Supplementary Figure 2.** Flow cytometric gating strategy to detect proliferating
T cells. Peripheral blood cells (1 * 10^6^ cells/ml) were stimulated with CD3/CD28 (100 ng/ml) or PHA (10 µg/ml). After 72 h, the T cells were stained with APC–H7 (mouse anti-human CD45 clone 2D1, 2.5 µl), FITC (mouse anti-human CD3, clone SK7, 5 µl,) and 7-Amino-Actinomycin D (7-AAD). Based on the following gating FSC vs. SSC **(A)** and CD45 vs. SSC **(B)** (mathematical connected by AND-operation) the T cells (CD3^+^) were separated in a third dot plot (CD3 vs. SSC) **(C)**. Live T cells were detected by dot plot (CD3 vs. 7AAD) **(D)**. The decrease of VPD450 dyes intensity in proliferating CD3^+^ cells **(E)**.
