# Supplementary material for: Evaluating STAT5 Phosphorylation as a Mean to Assess T Cell Proliferation
Source: Front Immunol. 2019 Apr 5;10:722. doi: 10.3389/fimmu.2019.00722 (PMC6460883; doi:10.3389/fimmu.2019.00722)
Supplement: Supplementary file 3 [file Data_Sheet_3.docx]

**
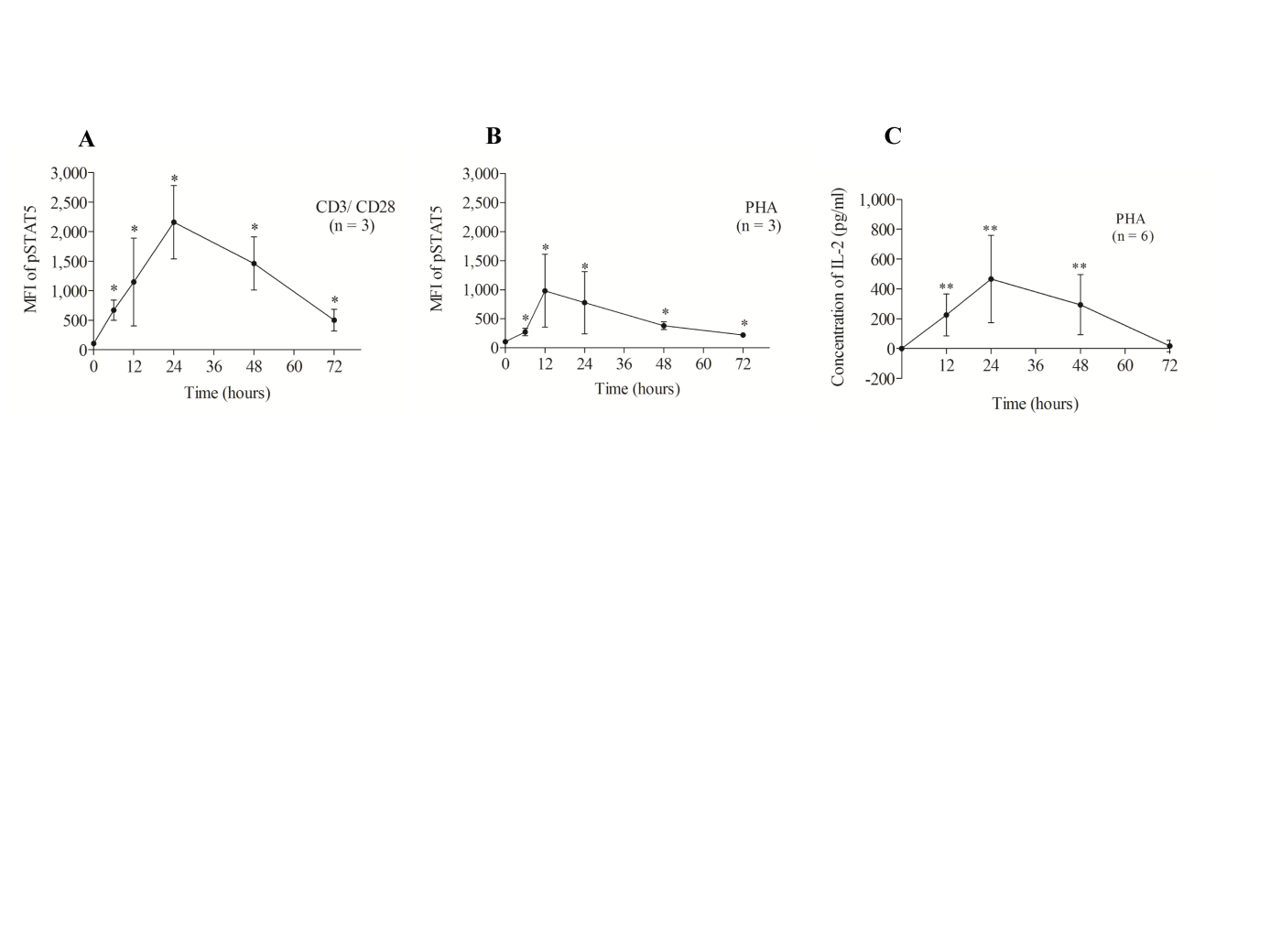
Supplementary Figure 3.** Time dependent phosphorylation of STAT5A and determination of IL-2 production. PBMCs (1 * 10^6^ cells/ml) were stimulated either with **(A)** CD3/CD28 (100 ng/ml) or **(B)** **(C)** PHA (10 µg/ml) for different times (0, 6, 12, 24, 48, 72 h). MFI of pSTAT5A was analyzed (**A)** and **(B)** and supernatants were collected and IL-2 concentration was determined by EIA **(C)**. Each point represents the mean ± SD; *, p < 0.05 **, p < 0.001; MFI, median fluorescence intensity.
